# Biocompatible Semi-Interpenetrating Materials Based on Poly(3-hydroxyalkanoate)s and Poly(ethyleneglycol) Diacrylate

**DOI:** 10.3390/gels8100632

**Published:** 2022-10-06

**Authors:** Laura Brelle, Agustin Rios de Anda, Teoman Ozturk, Nathalie Didier, Estelle Renard, Valérie Langlois

**Affiliations:** 1 Univ Paris Est Creteil, CNRS, ICMPE, F-94010 Creteil, France; 2 Univ Paris Est Creteil, INSERM, EFS, IMRB, F-94010 Creteil, France

**Keywords:** poly(3-hydroxyalkanoate)s, semi-interpenetrating networks, poly(ethylene glycol) diacrylate, biocompatible, gels

## Abstract

Biocompatible gels based on poly(3-hydroxyalkanoate)s (PHAs) were developed by radical polymerization in the presence of poly(ethylene glycol) diacrylate (PEGDA). In order to elaborate cross-linked networks based on PEGDA and PHAs, several PHAs were tested; saturated PHAs, such as poly(3-hydroxybutyrate-co-3-hydroxyhexanoate) (PHBHHx) or poly(3-hydroxyoctanoate) (PHO), and an unsaturated PHA, poly(3-hydroxyoctanoate-co-3-hydroxyundecenoate) PHOU. The PHA_x_PEGDA_1−x_ networks obtained in this work were studied by FTIR, Raman spectroscopy, DSC, TGA and NMR. The microscopic structure varied according to the mass proportions between the two polymers. Time Domain ^1^H DQ NMR measurements demonstrated that in the case of the unsaturated PHA, it was chemically crosslinked with PEGDA, due to the presence of double bonds in the lateral groups. The organogels were able to swell in organic solvents, such as THF, up to 2000% and in water up to 86%. It was observed by rheological analysis that the stiffness of the networks was dependent on the content of PHA and on the degree of cross-linking. The biocompatible characters of PHOU and PEGDA were not affected by the formation of the networks and these networks had the advantage of being non-cytotoxic to immortalized C2C12 myoblast cells.

## 1. Introduction

Poly(hydroxyalkanoate)s (PHAs) are naturally occurring aliphatic polyesters synthesized by many microorganisms when they are subjected to stress conditions using their specific intracellular enzymes [1,2,3,4]. PHAs are classified according to their side chain lengths: the short chain length PHAs (scl-PHAs) are composed of 4–6 carbon atoms, medium chain length (mcl-PHAs) and long chain length (lcl-PHAs) consist of 7 or more and over 15 carbon atoms, respectively. PHAs can be completely biodegraded by intra- or extracellular enzymes [5,6,7]. Their biodegradability [8,9,10] and biocompatibility properties allow them to be considered for a wide range of application areas such as environmental, medical, or pharmaceutical applications like drug delivery systems and tissue engineering [11,12,13,14,15,16,17].

However, since PHAs are very hydrophobic polyesters, copolymer synthesis, blends, and chemical modifications have been extensively studied [18,19,20,21,22,23,24], in order to enlarge their hydrophilicity. The combination with hydrophilic PEG or ionic moieties has been used to prepare amphiphilic block copolymers [25], graft copolymers [26,27] and water-soluble PHAs [26]. However, phase separation or poor interfacial adhesion between PHAs and the blends may limit the homogeneity at the molecular and mesoscopic scales of the obtained materials, thus influencing the polymer properties. Recently, Zhang et al. prepared organo/hydrogels via photo-cross-linking unsaturated PHA and PEG dithiol [28]. However, these gels were only formed by using unsaturated PHA. In this context, we proposed elaborating semi-interpenetrating polymer networks (semi-IPNs) by using saturated or unsaturated PHA to form gels. The formation of IPNs is defined as the combination of two independently cross-linked polymers with improved compatibility between the polymers. The formation of these networks allows a synergy of properties between the polymers involved in the formation of the material [29,30,31]. Interpenetrating and semi-interpenetrated networks based on PHAs prepared by reaction under UV radiation have already been described in the literature [32,33,34]. Biobased semi-IPNs, in which PHBHV is embedded in a network, have been developed to improve the mechanical properties of PHBHV. Semi-IPNs were obtained by cross-linking sunflower oil and trimethylolpropane tris(3-mercaptopropionate), TriSH. This study revealed an improvement of the deformability of PHBHV, due to the plasticization domains, by the sunflower oil– TriSH domains.

In this context, semi-IPNs based on different saturated and unsaturated PHAs and poly(ethylene glycol) diacrylate (PEGDA) added at different mass ratios were prepared and characterized. Samples were synthesized by photoirradiation in the presence of a photoinitiator, DMPA. PEGDA is a highly hydrophilic and biocompatible polymer [35] that has already been used to produce materials for biological applications, drug delivery [36], and tissue engineering [37]. The PHA_x_PEGDA_1−x_ networks obtained in this work were studied by FTIR, Raman spectroscopy, DSC, and TGA to assess the structures and properties of these materials. Furthermore, their network structure was analyzed by Time Domain Double quantum (DQ) ^1^H NMR experiments. By considering this technique, understanding the multiscale structure–properties relationship for these materials was ascertained, as this technique has proven to be relevant for the study of the intrinsic morphology and cross-linking density of cross-linked polymers [38,39,40,41,42,43]. Lastly, the biocompatibility of the different biomaterials produced was evaluated by analyzing the behavior of C2C12 myogenic cells grown on their surface by sequential multiplexing of CellTox™ Green Cytotoxicity Assay and CellTiter-Glo^®^ 2.0 Assay.

## 2. Results and Discussion

### 2.1. Preparation of Semi-IPNs Networks PHA_x_PEGDA_1−x_

**Figure 1:**
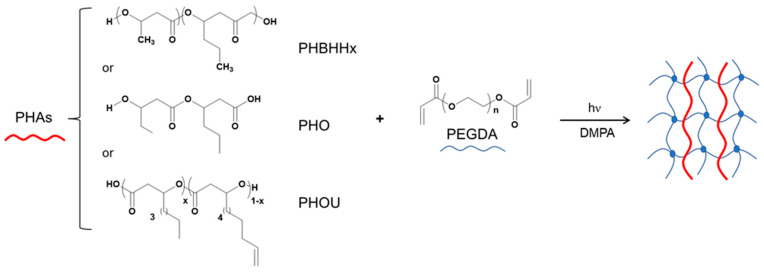
Elaboration of PHA_x_PEGDA_1−x_ networks under photoactivated cross-linking.

Different semi-IPN associating PHAs with PEGDA were elaborated by cross-linking under irradiation of PEGDA (Figure 1). PEGDA is a hydrophilic functional oligomer bearing two acrylate functions at each terminal end chain which are highly reactive in the presence of a photoinitiator. The PEGDA_100_ network was formed after 300 s of photoirradiation in the presence of DMPA. Semi-interpenetrating networks of PHA_x_PEGDA_1−x_ were prepared in the presence of PHBHHx, PHO, and PHOU in different proportions from 20 up to 80% to vary the flexibility of the obtained networks (Table 1).

**Figure 2:**
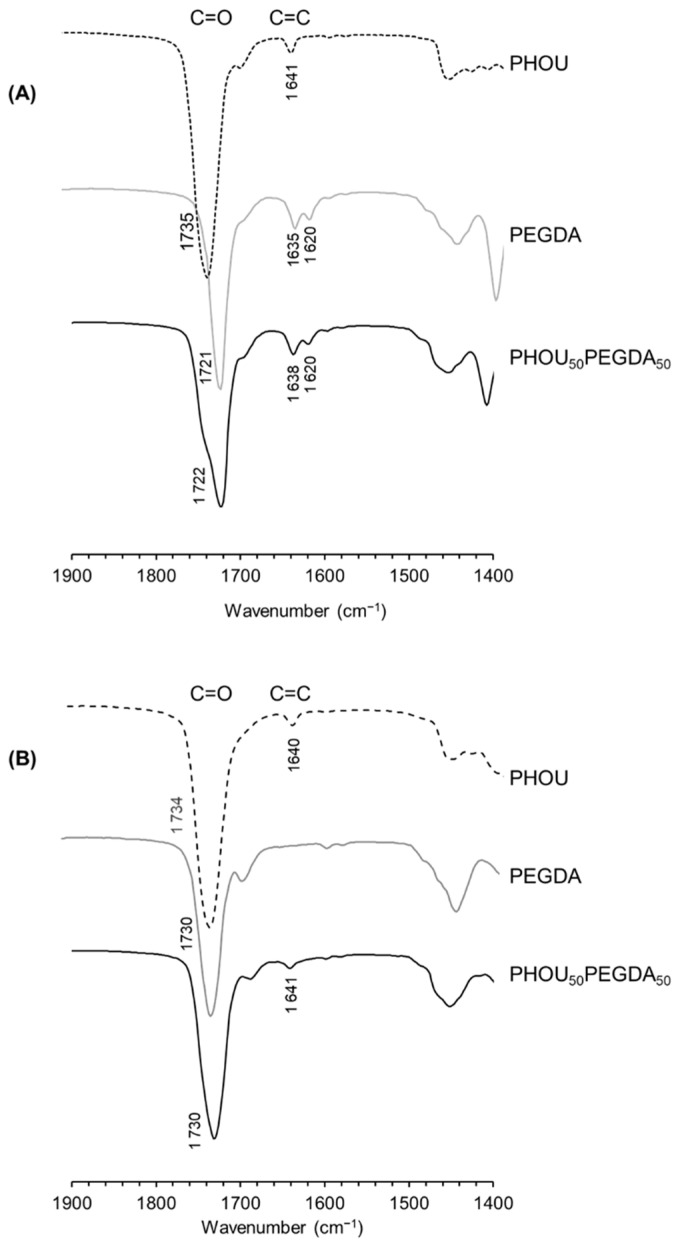
FTIR spectra of PHOU, PEGDA and PHOU_50_PEGDA_50_ before irradiation (**A**) and after irradiation (**B**).

The FTIR analysis showed that after polymerization of PEGDA, the intensity of the peaks at 1620 cm^−1^ and 1635 cm^−1^, characteristic of the double bonds, decreased sharply (Figure 2A). The double bond conversion rate was calculated by the ratio of the intensity of the ester bands at 1721 cm^−1^ and the intensity at 1620–1635 cm^−1^. After irradiation, the peaks characteristic of the double bonds disappeared almost completely and the conversion rate was close to 100% (Figure 2B).

By using saturated PHAs, such as PHBHHx and PHO, gels were formed when the concentration was equal to 100 g·L^−1^; although, in the case of PHOU, gels were formed when the PHOU concentration reached 40 g·L^−1^. This showed a difference in behavior between saturated and unsaturated PHAs during gel formation. Moreover, the soluble fractions of the networks formed with PHOU were inferior to 7 wt%, although they reached 30 wt% with the saturated PHAs.

The very low soluble content of free PHOU suggested that PHOU was retained by covalent bonds inside the network to form a real interpenetrating network. On the contrary, the networks formed with saturated PHAs only had a semi-interpenetrated network structure, because the saturated PHAs were not covalently bound to the PEGDA network. Although PEGDA and PHOU have double bonds, the FTIR results showed that their reactivities were very different. The conversion rate of the double bonds of PHOU was about 3% (Table 2). The unsaturations possessed by PEGDA were, thus, more reactive than the double bonds of PHOU. Moreover, the concentration of PHOU was further maintained at 100 g·L^−1^ to allow maximum cross-linking between PHOU and PEGDA and to reduce the homopolymerization of PEGDA (Table 2).

**Figure 3:**
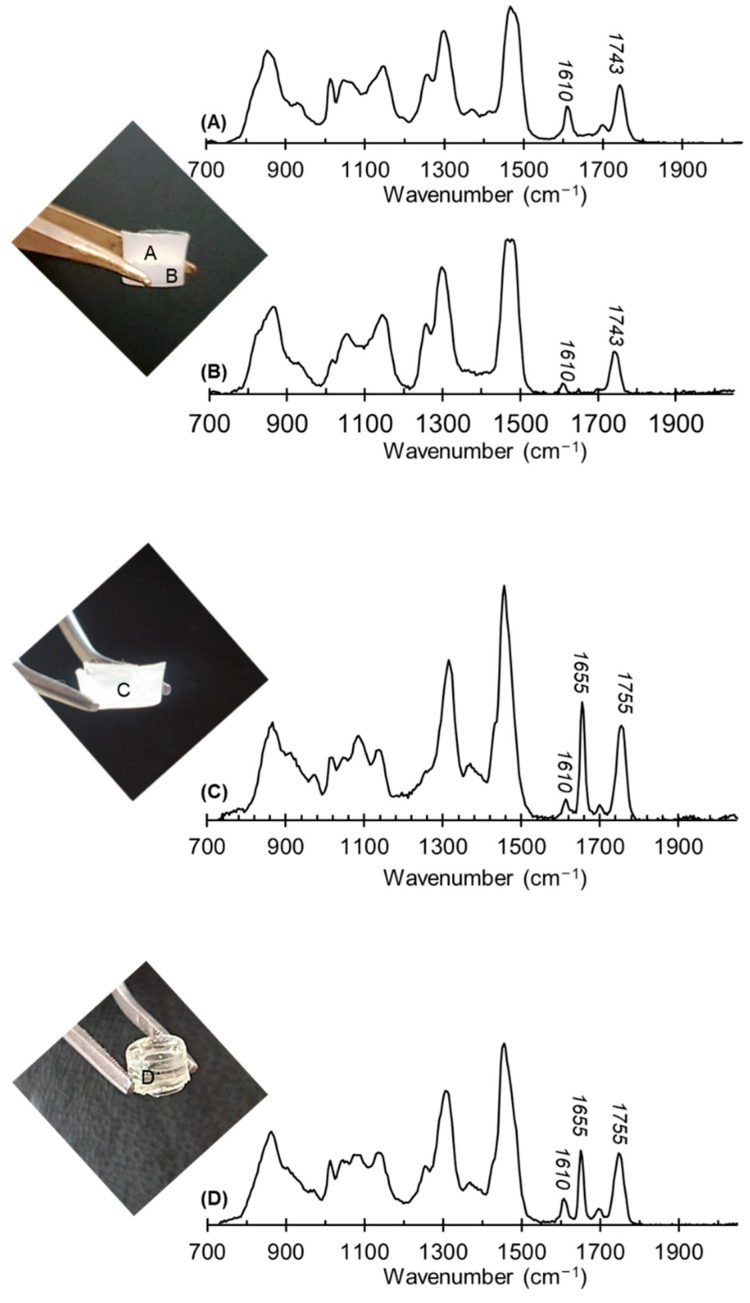
Raman spectra of the two zones (**A**,**B**) present in the PHBHHx20PEGDA80 network, PHOU_20_PEGDA_80_ network (**C**) and PHOU_50_PEGDA_50_ network (**D**).

Moreover, the networks based on PHBHHx_20_PEGDA_80_ were observed to not be homogeneous and presented very distinctive heterogeneous regions evidenced by Raman spectroscopy. The Raman analysis, highlighted by the presence of characteristic bands of each region, was performed (Figure 3), revealing one region rich in PEGDA (Figure 3A) and another rich in PHBHHx (Figure 3B).

The networks obtained with PHOU were found to be more homogeneous. The Raman spectra showed the presence of PHOU double bonds at 1655 cm^−1^ and PHOU ester groups at 1755cm^−1^ (Figure 3C,D). The presence of PHOU double bonds after the network formation showed that only a fraction of PHOU double bonds was consumed during network formation, but that this fraction was sufficient to chemically bind PHOU to the network and improve the homogeneity. For these reasons, we only selected the PHOU_x_PEGDA_1−x_ for further studies.

### 2.2. Characterization of Semi-IPNs, PHA_x_PEGDA_1−x_

**Figure 4:**
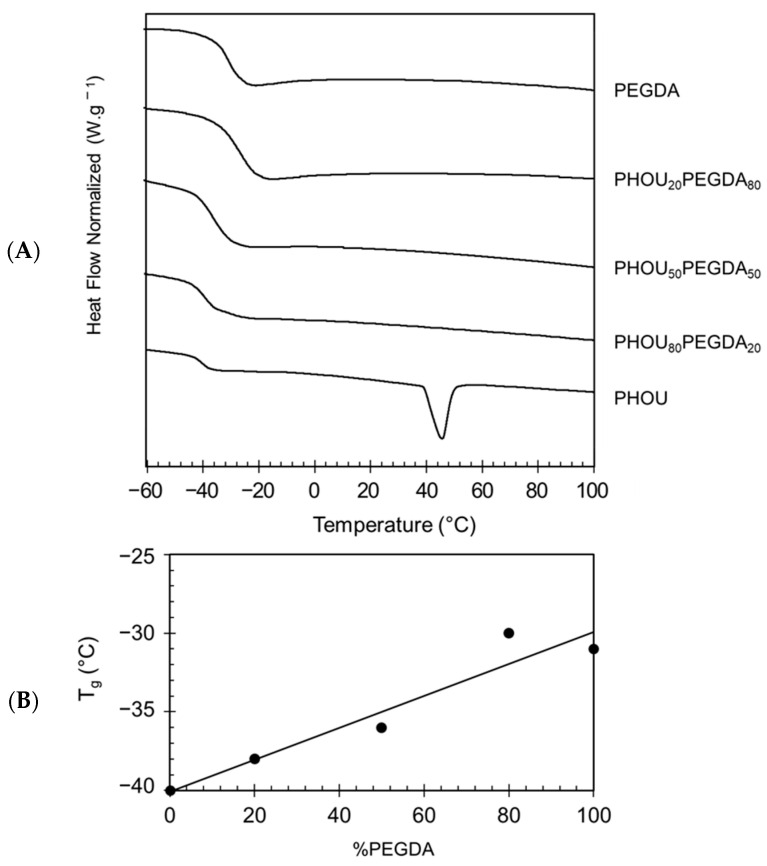
DSC thermograms of PHOUxPEGDA_1−x_ (**A**) and variation of T_g_ in function of PEGDA content (**B**).

The cross-linking prevented the crystallization of the PHOU, as was expected for cross-linked materials. This was evidenced by the disappearance of the melting temperature of the initial PHOU. Furthermore, the glass transition temperature T_g_ measured by DSC also lined up closely to the theoretical values determined by the Fox equation (Figure 4B), attesting to the homogeneity of the networks. This equation allowed a prediction to be made about the theoretical T_g_ values of two miscible polymers, considering which corresponded to the weight fraction of the two polymers. The increase of the PEGDA mass percentage within the network allowed increase of the T_g_ of the PHOU which, thus, increased from −40 °C for the PHOU alone to −28 °C for the PHOU_20_PEGDA_80_ network. This increase in T_g_ and the presence of a single peak might allow one to hypothesize that PHOU_x_PEGDA_1−x_ co-networks were formed.

(1) $$\frac{1}{T_{g}}=\frac{w_{\mathrm{PHOU}}}{T_{g_{\mathrm{PHOU}}}}+\frac{w_{\mathrm{PEGDA}}}{T_{g_{\mathrm{PEGDA}}}}\;\mathrm{Fox}\;\mathrm{equation}$$

**Figure 5:**
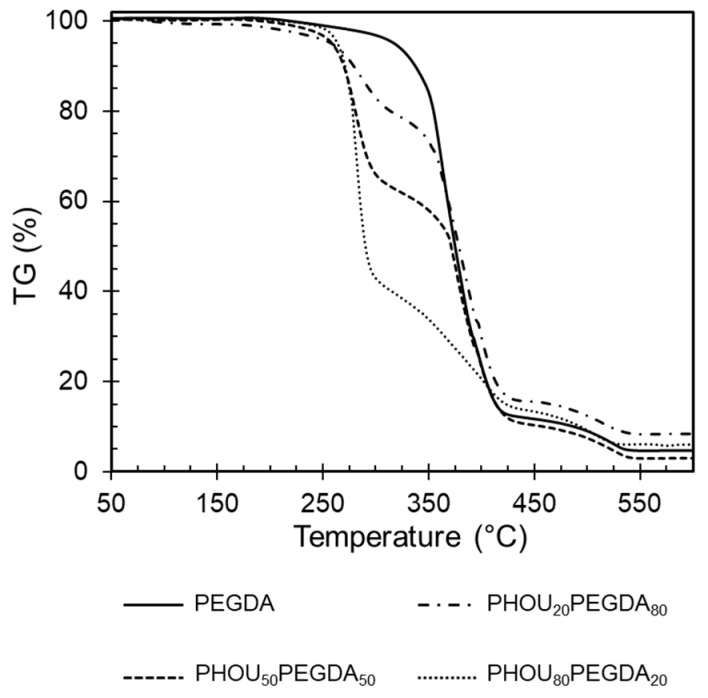
TGA spectra of PEGDA and PHOU_20_PEGDA_80_, PHOU_50_PEGDA_50_, PHOU_80_PEGDA_20_.

Figure 5 shows the TGA thermograms for the PHOU_x_PEGDA_1−x_ networks. T_d30_, temperature values at which 30% weight loss took place, were extracted from these thermograms, and are listed in Table 2. It was observed that PHOU and PEGDA decomposed from 250 °C to 300 °C and from 300 °C to 540 °C, respectively.

The decomposition of the different PHOU_x_PEGDA_1−x_ took place in two steps corresponding to the presence of each of these two components. However, it was observed that the thermal stability of the PHOU part increased. This might have been due to the presence of PEGDA within the network, as was attested to by the increase of the Td_30_ from 280 to 370 °C when the PEGDA content increased from 20 to 80 wt%. Furthermore, from the TGA thermograms, the mass content of PHOU linked in the networks were determined. It was observed that the PHOU amount was of the same order of magnitude as the initial mass composition, which confirmed that PHOU was partially linked by covalent bond to PEGDA. However, when the initial content of PHOU reached 80 wt%, only 55 wt% of PHOU content was determined in the network, attesting to the fact that some PHOU chains were not fully linked to the network. Nevertheless, the formation of these three-dimensional structures led to an overall increase of PHOU thermal stability.

### 2.3. Time Domain ^1^H DQ Solid State NMR

**Figure 6:**
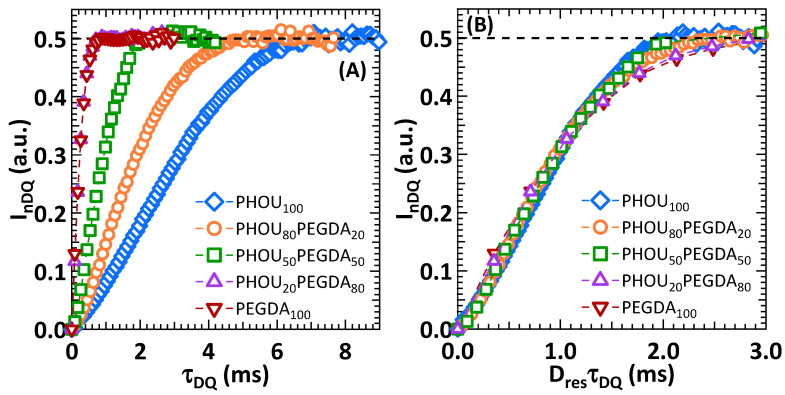
Normalized In_DQ_ ^1^H DQ NMR signals as a function of time τ_DQ_ (**A**) and normalized by D_res_τ_DQ_ (**B**) for all studied samples at T = T_g_ + 90 °C. D_res_ values are listed in Table 3.

The DQ build-up normalized I_nDQ_ signals were then obtained by ^1^H DQ NMR measurements for all samples. These signals are plotted as a function of the time τ_DQ_ in Figure 6A. To deepen and quantify these measurements, a numerical analysis was undertaken. ^1^H DQ NMR experiments gave access to the dipolar residual constant D_res_, which was proportional to the slope of the I_nDQ_ signal and was associated to an average local dynamic segmental orientation parameter. Most importantly, D_res_ was proportionally and directly related to the cross-link density ν_C_ (i.e., comprising both the chemical cross-links and physical entanglements) as described by Equation (2) [36,38,39]:

(2) $$k\frac{D_{\mathrm{res}}}{D_{\mathrm{stat}}}\propto \frac{1}{M_{C}}\propto v_{C}$$

where D_stat_ is the static dipolar coupling constant and k a proportionality factor related to the networks Kuhn length (i.e., distance between two chemical or physical nodes). In this work, the values for k and D_stat_ were not obtained as the numerical value of D_res_ gave a good insight into the material’s cross-link density. As such, D_res_ was obtained by fitting the corresponding I_nDQ_ signal in Equation (3):

(3) $$I_{\mathrm{nDQ}}=0.5\left[1-\exp{\left(-D_{\mathrm{res}}\tau_{\mathrm{DQ}}\right)}^{n}\right]$$

where n is the network homogeneity exponent varying between 1 and 2. The closer n is to the value of 2, the more homogeneous the networks are [38,39]. The numerical values of D_res_ and n are listed in Table 3, Figure 6A shows firstly that PHOU_100_ exhibited an I_nDQ_ signal, which meant that this polymer was indeed chemically cross-linked. Moreover, Figure 6A shows that the I_nDQ_ signal became steeper when the amount of PEGDA in the networks increased. This was confirmed by the D_res_ values listed in Table 3. This meant that the presence of PEGDA induced an increase in cross-link density v_C_ in the networks. To deepen this analysis, the obtained I_nDQ_ signals were normalized by D_res_τ_DQ_ so as to study the evolution of the network morphology. Such signals are plotted in Figure 6B.

Figure 6B shows that the I_nDQ_ signals for PHOU_100_, PHOU_80_PEGDA_20_, and PHOU_50_PEGDA_50_ superposed well with each other. This was also the case for PHOU_20_PEGDA_80_ and PEGDA_100_, although these latter I_nDQ_ signals did not superpose to the former. This would imply that the PHOU_100_, PHOU_80_PEGDA_20_, and PHOU_50_PEGDA_50_ networks possessed a similar chemical and physical network morphology, that is they exhibited similar chemical reactions leading to a given physical network organization. The same could, thus, be concluded for the PHOU_20_PEGDA_80_ and PEGDA_100_ networks. These observations were highlighted when the D_res_ numerical values were plotted as a function of PEGDA content in the networks, as well as by the values of the homogeneity factor n listed in Table 3.

**Figure 7:**
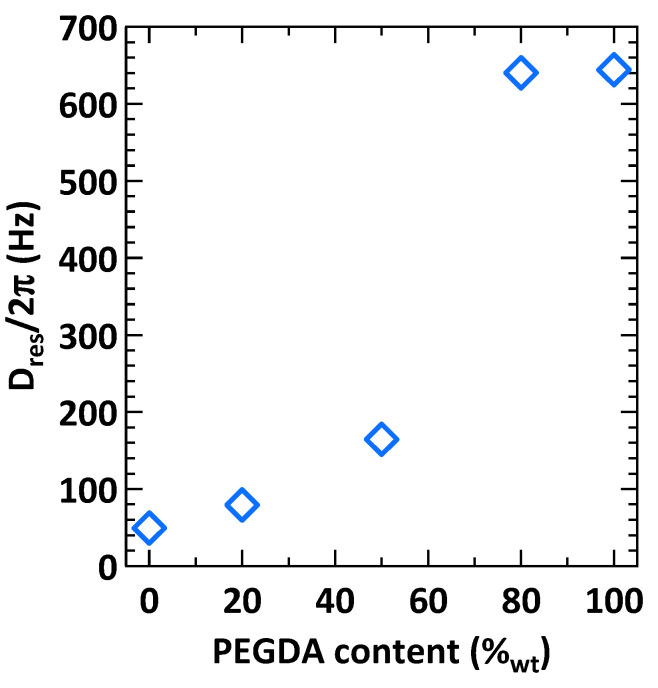
D_res_ values obtained from ^1^H DQ NMR measurements as listed in Table 3 plotted as a function of PEGDA content.

Figure 7 shows that when the PEGDA content in the networks was below 50 wt%, the D_res_ values, and thus the cross-link density v_C_, increased slightly. For such networks, their morphology was defined by the PHOU component. However, when the PEGDA content became majoritarian, the cross-link density increased abruptly and the network morphology was controlled by this moiety. Finally, Table 3 shows that the homogeneity factor n diminished with PEGDA content. This meant that the networks became more heterogeneous, which would be a signature that PHOU and PEGDA formed semi-interpenetrated networks (semi-IPN). Indeed, semi-IPN tended to be very heterogeneous at the molecular scale, as they represented a mixture of polymers with very different chemical structures.

### 2.4. Rheological Properties and Swelling Behavior of Networks

The mechanical properties of the different networks were studied by rheology measurements. The elastic modulus G′, characterizing the rigidity of the material, was found to be higher than the viscous modulus G″ over the whole angular frequency for an oscillatory stress of 20% or 50%. This trend showrf that all the obtained materials had a gel-like behavior with remarkable mechanical properties, with elastic moduli varying from 1088 Pa to 8853 Pa (Table 4).

It was observed that the rigidity increased with the mass content of PEGDA, which increased the degree of cross-linking of the grafting, leading to higher mechanical properties. The presence of cross-linked or free PHOU chains within the network could also induce a certain flexibility to the network. Indeed, free chains that were sufficiently long and could move freely lessened the rigidity provided by the presence of cross-links.

**Figure 8:**
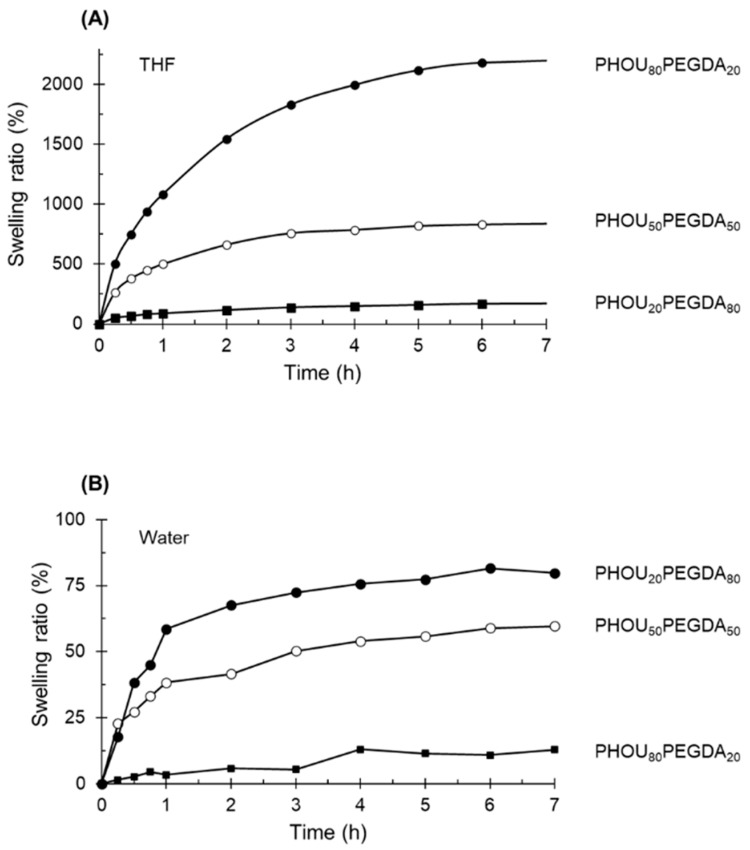
Swelling behaviors of the different networks in THF(**A**) and water (**B**).

The swelling kinetics of the networks were then studied in THF. All networks had the capacity to swell in THF which had a good affinity with PHOU. However, the behavior depended on the content of PHOU. For a PHOU_80_PEGDA_20_, the swelling rate after 6 h reached 2178% (Figure 8). This was in contrast with PHOU_20_PEGDA_80_ which only slightly swelled in THF, reaching 168% after 6 h. Thus, the networks formed from 80 wt% PHOU had a better affinity with THF, which resulted in a higher swelling rate. However, concerning the swelling in water, the trend was found to be reversed: the higher the proportion of PEGDA, the higher the swelling rate in this solvent was. This was due to the hydrophilicity of PEGDA. For the PHOU_20_PEGDA_80_ networks, the swelling percentage reached 82% after 7 h. The network formed from PHOU_80_PEGDA_20_ swelled only very slightly, reaching a swelling rate after 7 h of 13%. The swelling rate was, therefore, inversely proportional to the degree of cross-linking of the network. From these characterizations, it can be stated that the ability of networks to swell in an organic solvent, such as THF, allows them to be defined as organogels.

### 2.5. Biological Test

**Figure 9:**
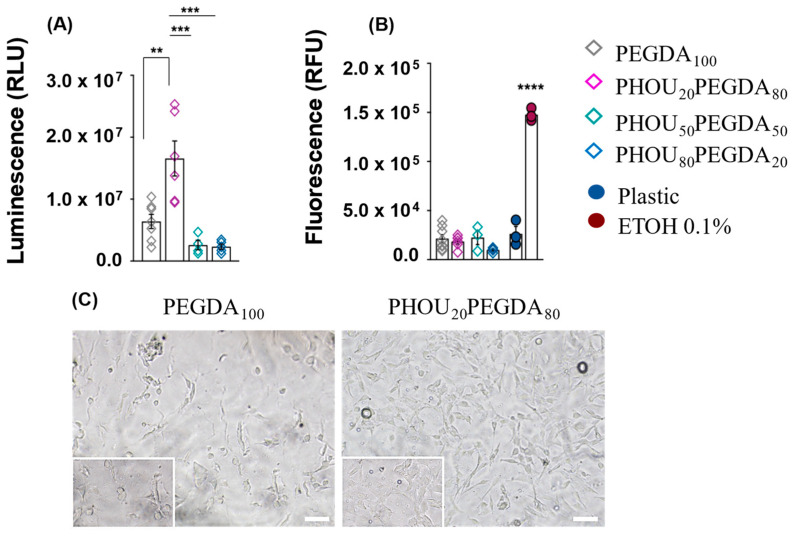
Evaluation of PHOUxPEGDA_1−x_ biomaterial biocompatibility. C2C12 cells were seeded at the same density (3000 cells/cm^2^) on 1 cm^2^ biomaterials and expanded for 5 days in Growth Medium. Measurement of luminescence (RLU) associated with viability of the cells (**A**) and of fluorescence (RFU) associated with cytotoxicity after expansion on the biomaterials (**B**). C2C12 cells incubated with 0.1% of ethanol diluted in GM for 30 min were used as positive control of toxicity. C2C12 cells expanded on plastic dish were used as negative control. Data are represented as the mean ± s.e.m of 4 to 7 independent experiments. Statistical significance was determined by One way ANOVA tests followed by Tukey’s multiple comparisons test, with ** *p* < 0.01, *** *p* < 0.001 and **** *p* < 0.0001. (**C**) Representative photomicrons showing C2C12 cell morphology and density after 5 days of culture on PEGDA_100_ and PHOU_20_PEGDA_80_. Inlets show higher magnification. Scale bars: 50 µm.

The biocompatibility of the different biomaterials was assessed by comparing the viability of C2C12 myogenic cells after 5 days of culture (Figure 9). This luminescent cell viability assay is based on ATP content, a required co-factor of luciferase reaction. The luminescence produced is, thus, proportional to the amount of ATP present reflecting cellular metabolic activity. Luminescence intensity (Relative Luminescence Units) was significantly increased when cells were cultured on PHOU_20_PEGDA_80_ biomaterial compared to PEGDA_100_, PHOU_50_ PEGDA_50_ and PHOU_80_ PEGDA_20_ biomaterials. In addition, similar luminescence intensity was obtained when cells were amplified on PEGDA_100_, PEGDA_50_ or PHOU_80_ PEGDA_20_. These data showed that after 5 days of culture, a higher number of viable cells were present on PHOU_20_PEGDA_80_ biomaterial than on PEGDA_100_ or on biomaterials containing higher proportions of PHA (i.e., PHOU_50_ PEGDA_50_ and PHOU_80_ PEGDA_20_) (Figure 9A).

We, therefore, assessed potential cytotoxicity of PHOU_x_ PEGDA_1−x_ biomaterials by measuring fluorescence intensity (Relative Fluorescence Units) corresponding to the binding of CellTox™ Green dye to the DNA of cells with impaired membrane integrity (Figure 9B). C2C12 cells exposed for 30 min to growth medium containing 0.1% ethanol were used as a positive control of toxicity, and cells grown under standard conditions on a plastic dish were a negative control. The fluorescence intensity was significantly higher when cells were treated with 0.1% ethanol compared to all other conditions, whereas no difference was observed between cells grown on plastic, PEGDA_100_ or PHOU_x_PEGDA_1−x_ biomaterials. Thus, the reduced number of cells obtained after culture on PHOU_50_PEGDA_50_ and PHOU_80_PEGDA_20_ biomaterials was not due to a cytotoxic effect of increasing proportions of PHA but rather to a lack of proliferation or a poor adhesion of the cells. Additionally, we assessed cell morphology and confluency by optical microscopy (Figure 9C). After 5 days of culture, we observed that the cells amplified on PHOU_20_PEGDA_80_ biomaterial showed the characteristic elongated shape of C2C12 cells and a high cell density. Conversely, we noted the presence of rounded cells on PEGDA_100_ biomaterial consistent with a poor cell adhesion as well as a low cell density. We, therefore, concluded that PHOU_20_PEGDA_80_ enabled better cell adhesion than PEGDA_100_ biomaterial which at least partly contributed to the increased cell proliferation observed. Overall, these experiments revealed that PHOU_20_PEGDA_80_ biomaterials are suitable for cell expansion and could, therefore, be considered as scaffolds for tissue engineering and regenerative medicine applications. Currently there is no suitable medium for the amplification of muscle stem cells called satellite stem cells. In this study, the multiplication capacity of immortalized C2C12 myoblastic cells was tested on PHOU_x_PEGDA_1−x_ supports. These C2C12 cells are less fragile than satellite cells. However, these first results are, thus, an open door to the development of supports for satellite stem cells.

## 3. Conclusions

Interpenetrating networks based on PEGDA and PHAs were formed by using saturated PHAs, namely PHOHHx and PHBHHx, and unsaturated PHOU. The results showed that the development of homogeneous and translucent networks was only possible in the presence of PHOU. The networks based on PHBHHx and PHOHHx presented two phases macroscopically, because PHBHHx and PHOHHx only entangled through the PEGDA network. The presence of unsaturation on the side chain of PHOU was, therefore, essential to obtain a homogeneous network because PEGDA chains could be grafted to the PHOU. PHOU was, therefore, the most suitable PHA for the elaboration of homogeneous network with PEGDA. This was shown with the disappearance of the PHOU melting temperature and the presence of a single T_g_ which increased with the PEGDA content. Time Domain ^1^H DQ NMR measurements demonstrated that PHOU was chemically cross-linked with PEGDA. Moreover, this technique showed that the morphology of these networks depended on the PEGDA content and that when it increased the networks became more heterogeneous, which could be a signature of obtaining semi-IPN materials. The network with the highest mass proportion of PEGDA, i.e., 80%, had the best swelling rate in water in relation to its hydrophilicity and a higher G′ modulus, due to its rigidity. Since we observed that PHOU_20_PEGDA_80_ biomaterials supported the expansion of myogenic cells, we anticipate that they could be further used in regenerative medicine as scaffolds to promote muscle remodeling in massive lesions (VML) [44].

## 4. Materials and Methods

### 4.1. Materials

Poly(3-hydroxybutyrate-co-3-hydroxyhexanoate) (PHBHHx), containing 20% of hexanoate units (Mw = 69,000 g·mol^−1^, Ip = 2.2), was provided by Georges CHEN from the Center for Synthetic and Systems Biology of Tsinghua University. Poly(3-hydroxyoctanoate-co-3-hydroxyhexanoate) (named PHO for ease of reading) and Poly(3-hydroxyalkanoate-co-3-hydroxyundecenoate), PHOU with 32% of double bonds (Mw = 185,000 g·mol^−1^) were kindly provided from M. Zinn (HES-SO Valais-Wallis—Haute Ecole d’Ingénierie, Sion, Switzerland). Poly(ethylene glycol) diacrylate (Mn = 575 g·mol^−1^ (PEGDA_575)_)) and 2,2 dimethoxy-2-phenylacetophenone (DMPA, Irgacure 651) were purchased from Sigma-Aldrich. Tetrahydrofuran (THF) was provided from Carlo Erba. They were used as received without prior purification.

### 4.2. Synthesis of PHA_x_/PEGDA_y_

Various solutions of PHAs (PHBHHx, PHO, PHOU) at 100 g·L^−1^ in THF were prepared. 5 wt% of photoinitiator, DMPA was solubilized in 0.5 mL of PHAs solution, after vortex agitation 30 s. was added to make a material with different weight proportions, 20/80, 50/50 and 80/20 (PHA_x_PEGDA_1−x_). To obtain a homogeneous mixture, the solutions were agitated 30 s with a vortex agitator. The solutions were then left to rest in a 2 mL vial covered by a polypropylene film, (which did not absorb radiation), maintained with an elastic band. The polymerization between PHAs and PEGDA was carried out in the presence of DMPA. Each mixture was placed at 9 cm from the UV source and was irradiated at 100% of the total lamp power for 300 s at room temperature, without stirring, with a mercury xenon lamp (180 mW·cm^−2^) Lightning cure LC8 (L8251) lamp from Hamamatsu coupled with a flexible light guide. The formed network was then removed from the mold and rinsed in 6 mL of THF for 2 h to eliminate free polymer chains. It was then immersed in a large quantity (*ca.* 20 mL) of THF/H_2_O (with *v/v* 0.1/1) mixture for 2 h. This protocol was used to avoid chains of highly hydrophobic PHAs coming into direct in contact with water molecules after formation. The THF wash solution was recovered and evaporated in the fume hood until complete evaporation. The pills were then weighed and ^1^H NMR in CDCl_3_ was performed.

### 4.3. Characterization

#### 4.3.1. Preparation of Semi-IPNS Networks PHA_x_PEGDA_1−x_

The molar mass of each semi-IPN was determined at 25 °C by size exclusion chromatography (SEC), with chloroform as the eluent, at a flow rate of 1 mL·min^−1^. Measurements were done with a Schimadzu LC-10AD pump with two Shodex GPC K-805L columns (5µm Mixte-C) at a concentration of 10 mg·mL^−1^ connected in series on a Wyatt Technology Optilab Rex interferometric refractometer, used as a detector. Low polydispersity index polystyrene (3 × 10^4^–2 × 10^6^ g·mol^−1^) were used as standards.

To evaluate the conversion rate of double bonds, FT-IR spectra, normalized on peak at 1722 cm^−1^ corresponding at C=O function, were recorded on a Brucker Tensor 27 spectrometer with 32 scans equipped with an ATR apparatus at room temperature.

The chemical structure was analyzed by using a Horiba Xplora confocal Raman microscope (Horiba Jobin Yvon, Arcueil, France). An excitation wavelength at 633 nm was provided by a He-Ne laser. Spectra were recorded in the 4000–400 cm^−1^ wavelength range with microscope lenses x50 long-working distance (Long Working Distance M Plan Semi-Apochromat, LMPLFL50x, Olympus, Shinjuku, Tokyo, Japan) and 10x (M Plan Achromat, MPLN10x, Olympus), in the dark at 25 °C and in air. The acquisition of the spectra was done with the help of the LabSpec software (Horiba Scientific, Edison, NJ, USA). Exposure time depended on the quality of the spectrum. The accumulation and acquisition were five times and 5 s, respectively.

#### 4.3.2. Thermomechanical Behavior of Networks PHA_x_PEGDA_1−x_

The thermal and decomposition (TGA) characteristics of different semi-IPNs were determined on a Setaram Setsys Evolution 16 apparatus by heating the samples at a rate of 10 °C.min^−1^ from 20 to 600 °C under atmospheric air and normalized to the C=O band at 1732 cm^−1^.

Differential scanning calorimetry (DSC) measurements were recorded on TA Instruments DSC25. A first heating run from −80 °C to 200 °C with a heating rate of 10 °C/min was performed to determine the melting temperature and the fusion enthalpy. This was followed by a cooling run to −80 °C with a cooling ramp of 200 °C/min. The T_g_ was obtained in a second heating run from −80 °C to 200 °C at 10 °C/min.

#### 4.3.3. Time Domain ^1^H DQ Solid State NMR

Time Domain ^1^H Double Quantum DQ measurements were carried out on a Bruker Avance III 400 NMR equipped with a 5 mm ^1^H solenoid static probe. DQ measurements were based on Baum-Pines pulse sequences [38] optimized by Saalwächter [39]. For these experiments to be able to access the network structure, they had to be conducted at a temperature where the molecular motions probed by this technique must be effective in the fast motion regime as has been previously described [42]. For most polymers this truly elastic domain is found at temperatures equal or above T_g_ + 90 °C, and, as such, all of the samples were studied at this temperature, ensuring that they were all tested at the same state of molecular dynamics in the temperature-independent regime of ^1^H DQ NMR measurements. Samples were finely cut then put in glass tubes and inserted in the 5 mm ^1^H solenoid static probe, where they were gradually heated up to T = T_g_ + 90 °C. The temperature was stabilized for one hour before conducting the measurements. The NMR spectra were then treated to obtain a DQ build-up normalized signal I_nDQ_ for each sample following the same approach that has been detailed in previous works [42].

#### 4.3.4. Rheological Properties and Swelling Behavior of Networks PHA_x_PEGDA_1−x_

The rheological properties were determined with a hybrid rheometer (TA Instruments AR1000) using a 20 mm planar geometry system with a 1° conical geometry. Tests were performed at 25 °C in a controlled temperature room, with evaporation of the water contained in the semi-IPNs limited by the presence of a solvent trap. Frequency sweeps were undertaken between 0.1 and 100 rad·s^−1^ for an applied deformation of 20% to obtain the values of the viscoelastic moduli G′ (storage modulus or elastic modulus) and G″ (loss modulus or viscous modulus).

### 4.4. Biological Tests

Biomaterials were sterilized by exposure to UV lamp for 1 h, transferred in 24-well culture plates and washed for 24 h in Growth Medium (GM) containing Dulbecco’s modified Eagle medium (DMEM, Gibco, New York, NY, USA), 20% Fetal Bovine Serum and 0.5% antibiotics for 24 h. The surface of the biomaterials was coated with gelatin 0.1% to optimize cell adhesion. C2C12 cells were seeded on biomaterials at a density of 3000 cells/cm^2^ in a small volume of GM for 2 h at 37 °C. Then, GM was added to fill the wells and the cells were amplified for 5 days at 37 °C under 5% CO_2_. The medium was renewed every 2 days. The cell-seeded biomaterials were then transferred to another plate, washed with HBSS (Gibco), and incubated with Trypsin for 10 min at 37 °C for the cells to detach. Cells were recovered with 1 mL of GM and pelleted by centrifugation at 300× *g* for 5 min. Cells were then transferred in opaque 96-wells plates (#30122300, Tecan, Männedorf, Switzerland). The toxicity of the biomaterials and the number of viable cells were determined by sequential multiplexing of CellTox™ Green Cytotoxicity Assay and CellTiter-Glo^®^ 2.0 Assay (#G9241, Promega, Madison, WI, USA) following the manufacturer’s instructions. Fluorescence (RFU) and luminescence (RLU) intensities were recorded using an Infinite^®^ 200 PRO multimode plate reader (Tecan). For optical analysis of living cell morphology, images were acquired using a Zeiss Axio Observer D1 inverted microscope.

## Figures and Tables

**Table 1:** Gel formation of PHA_x_PEGDA_1−x_ networks after 300 s of photoirradiation in the presence of DMPA and PHAs at 100 g·L^−1^.

Compositions	Aspect	Soluble Extract (%)
PEGDA_100_	Solid	0
PHBHHx_20_ PEGDA_80_	Solid	30.0 ± 5.0
PHBHHx_50_ PEGDA_50_	Liquid	nd
PHO_20_PEGDA_80_	Solid	24.0 ± 3.0
PHO_50_PEGDA_50_	Liquid	nd
PHOU_20_ PEGDA_80_	Solid	4.9 ± 0.5
PHOU_50_ PEGDA_50_	Solid	4.1 ± 0.2
PHOU_80_ PEGDA_20_	Solid	6.9 ± 3.7
PHOU_100_	Liquid	nd

nd: Abbreviation of no data.

**Table 2:** Characteristics of the networks.

Samples	Tconversion C=C ^(a)^ (%)	T_g_ ^(b)^ (°C)	T_m_ ^(b)^ (°C)	Td_30_ ^(c)^ (°C)
PEGDA_100_	99 ± 1	−30	-	371
PHOU_20_ PEGDA_80_	85 ± 8	−28	-	370
PHOU_50_ PEGDA_50_	70 ± 2	−36	-	301
PHOU_80_ PEGDA_20_	80 ± 2	−38	-	279
PHOU_100_	3 ± 1	−40	46	279

(a) determined by FTIR; (b) determined by DSC; (c) determined by TGA.

**Table 3:** D_res_ and n values obtained by ^1^H DQ NMR for the studied networks.

Samples	D_res_/2π (Hz)	N
PHOU_100_PEGDA_0_	49.3	1.68
PHOU_80_PEGDA_20_	79.5	1.55
PHOU_50_PEGDA_50_	164.4	1.57
PHOU_20_PEGDA_80_	640.1	1.32
PHOU_0_PEGDA_100_	644.2	1.25

**Table 4:** G′ and G″ values of networks at 10 Hz.

Samples	G′ (Pa)	G″ (Pa)
PHOU_20_PEGDA_80_	8853	388
PHOU_50_PEGDA_50_	7602	442
PHOU_80_PEGDA_20_	1088	145

## Data Availability

Not applicable.
